# Patient and public involvement in health literacy interventions: a mapping review

**DOI:** 10.1186/s40900-017-0081-z

**Published:** 2017-12-20

**Authors:** Stephanie Howard Wilsher, Julii Brainard, Yoon Loke, Charlotte Salter

**Affiliations:** Norwich Medical School, University of East Anglia, Norwich, NR4 7TJ UK

**Keywords:** Health literacy intervention research, Older people, Patient and public involvement, Mapping review

## Abstract

**Plain English summary:**

If people can read, understand and act on health information to better their health and reduce illness, they are thought to have “adequate” health literacy. Poor health literacy can mean people are less able to access health care and manage their health. Health literacy tends to worsen as adults get older, and is especially poor in adults age 65 and over. Ideally, health literacy interventions target people before age 65, to establish good skills and habits before people have many health problems associated with ageing. It is also good if researchers consult ordinary people, including patients and the public (PPI) when planning a programme to try to improve health literacy. This may help ensure individual needs are catered for.

We therefore looked for studies that described any role of patient or public representatives in the research planning stages. We explored how the representatives contributed to each project. We found only 20 studies that included people other than the research team. Lack of reporting and consultation with patient and public representatives may contribute to less success when public health programmes are undertaken.

**Abstract:**

**Background:**

Health literacy is the ability to understand, access and use health care and is a critical mediating factor that affects the health of older adults. Patient and public involvement in health and social care research, policy and design of care delivery is one mechanism that can promote production of better health literacy. This mapping review looks for and describes practices, concepts and methods that have been reported involving patients and public in the development and design of health literacy interventions for older people.

**Methods:**

Studies for the present review were selected from an inventory of health behaviour studies published between 2003 and 2013. The inventory was created by systematic searches on bibliographic databases (Medline, CINAHL, Scopus, Google) for health literacy interventions involving older people (50+ years) and resulted in screening of 5561 articles, of which 1097 met study inclusion criteria. For the research described in this article 96 of the 1097 studies specifically focused on health literacy and were independently screened by two reviewers to assess involvement of stakeholders other than investigators and participants.

**Results:**

Twenty studies included patient and/or public involvement in at least one research domain: design, management or evaluation. Involvement included volunteers, older people, patients, and/or community representatives.

**Conclusions:**

Patient and public involvement were rarely reported in studies on health literacy interventions for older people. Future intervention development needs high quality PPI, which is well reported to develop the evidence base and inform practice.

## Background

Health literacy, the ability to read, understand and act on health information, is a key determinant for improving world health [1, 2]. At its simplest, health literacy may mean the ability to take medications correctly or perform basic self-care after injury or during mild illness; more advanced health literacy is required for effectively managing a chronic condition like diabetes or rheumatoid arthritis. Therefore, health literacy is a critical mediating factor that affects patients’ ability to take part in their healthcare [3]. Health literacy is low for between 40 and 50% of the population in developed nations [4–6]. This has undesirable effects on a wide-range of health indicators and outcomes, and may impose additional costs of 3–5% onto total national health care budgets [7]. Efforts to increase health literacy in patients have been endorsed by governments [8–10] and professional associations [11–13]. The undesirable consequences of low health literacy are internationally recognised, leading to the creation of many national commissions and comprehensive reviews in the hunt for effective intervention strategies to improve health literacy [14–16].

Adults age 65+ are an especially vulnerable group, with regard to poor health literacy, for many (sometimes complex) reasons [17]. Cognitive decline, less educational attainment, and many years since acquisition and use of numeracy and literacy skills, co-occurring at a time of life when health problems may sharply increase and become more complex, are all possible factors in accelerating health literacy decline among older adults. There is increasing awareness in Europe that more should be done to ensure effectiveness of interventions to improve health literacy and thus health in older adults [18].

Broadly speaking, patient and public involvement (PPI) in health and social care research aims to encourage co-production of health care by giving non-investigators a voice in aspects of research design. Patient and public involvement refers to active partnership between patients and/or members of the public and researchers [19] to prioritise, design, manage, undertake and disseminate research. It is distinct from acting as a participant or a co-investigator in the research. Originating in the 1950s, PPI evolved as a challenge to ‘the unquestioned authority of medicine from health service users’ [4, 20]. Following accumulation of evidence regarding the worth of PPI [21], it has been implemented in Europe, the United States, Canada, and Australia. Support for PPI in the USA is led by Patient-Centred Outcomes Research Institute (PCORI) where patients can review and prioritise research projects [22]. Since 2013, NHS commissioners in the UK have a statutory duty to promote PPI in all aspects of research.

PPI in research can identify relevant questions to ask and outcomes to assess that are important to them and address their needs in a suitable fashion. For example, patients might not understand the health information they are given, especially if not culturally specific, or they may wish to know how best to live with a chronic condition rather than what is the “best” treatment. In so doing, it should be possible to move away from health and social care research that is *done to* people to that being *done with* people [23]. However, as yet, few clinical trials include patient-reported outcomes [24]. Health literacy broadly covers knowledge, behaviour and health outcomes that differ at the individual or group level. Using PPI in health literacy interventions should, therefore, be key to improving health outcomes.

This mapping review attempts to find and describe PPI in a sample of health literacy interventions. The purpose of a mapping review is to categorise studies and types of investigation and explore linked concepts used in a body of related research [25]. First, we implemented a customised search strategy to try to find reports on health literacy interventions that might include PPI. Then we read articles carefully, selecting for further descriptive analysis, articles that reported PPI activity. We describe PPI features that were found in the eligible articles. The results are described qualitatively but we do not treat these observations as definitive.

## Methods

### Data sources and selection criteria

Previous work by the authors [26] created an inventory of health behaviour studies published in 2003–2013, with the aim of developing policy and practical guidelines for health literacy promotion in Europe. The inventory was originally created by searching bibliographic and grey literature databases and sources (Medline, CINAHL, Scopus, Google) using a broad range of search terms related to health literacy and self-efficacy skills, for all interventions which included any older adults (although explicit targeting of this age group was not essential; “older adult” = age 50+). Age 50 has been suggested as a reasonable threshold for European public health research on older adults [27]. To create the inventory, 5561 articles were screened independently by two researchers; inclusion was confirmed by a senior academic with experience in health literacy research. 1097 articles were included in the inventory from the initial eligibility criteria, which were the inclusion of older adults in health literacy or compatible interventions [26]. For the research described in this article, we screened the title and abstract of these 1097 articles contain the exact phrase “health literacy” (*n* = 96). The abstracts of these 96 studies were duplicate screened for descriptions of studies that seemed to possibly describe involvement of stakeholders other than patients and investigators. The full texts of any abstracts that could not be excluded were read to search for the involvement of non-investigator patients, carers, community or charitable bodies in any aspects of research design including design of delivery, monitoring of evaluation (NIHR, 2014). Studies were excluded if it was not possible to discern that any of these groups were involved in any aspect of the research process. Among the remaining studies that described PPI involvement, we next posed two questions:

- Who is involved in the research?
- When and how are they involved?

### Analysis

We categorised patient and public involvement (PPI) within each study. Data were extracted for the number of people involved in each stage of the research process, who was involved and how were they involved. Following published guidance on possible PPI roles [28], we devised a taxonomy for PPI opportunities to input to the research process, designating the contribution domains (see Table 3) as follows: Identification and prioritisation of research objectives; (other aspects of) Design; Grant development; Project management or undertaking; Analysis and interpretation; Dissemination; Monitoring or evaluation. Two experienced qualitative researchers (SHW & CS) independently extracted data with differences resolved by a balanced discussion. There was an option to refer to a third investigator for very difficult decisions.

## Results

**Fig. 1:**
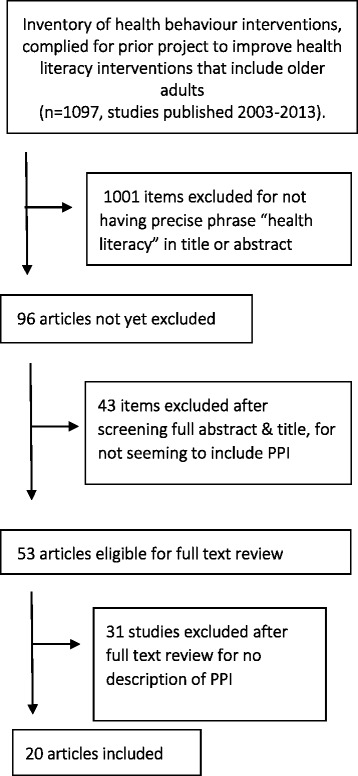
Flow diagram of study selection for the mapping review of Patient and Public Involvement

The study selection process is shown in Fig. 1. Of the 96 studies that mentioned health literacy, 43 were removed due to no suggestion of PPI in the title or abstract. Fifty-three studies were read in full and 20 studies fulfilled the inclusion criteria (evidence of PPI reported). Details of studies are shown in Table 1, with description of PPI**.**

**Table 1:** Descriptive details of patient and public involvement in included studies (see references [25–44])

Authors/date/ country	Title; study format	Who was involved, how many participants and how they participated
Age UK 2007–2012 UK	Fit as a Fiddle; single arm	Stakeholders (n = unclear) Design, management, evaluation Volunteers (*n* = 4500) Management of classes and supporting roles Older people (*n* = 881) Evaluation
Aspinall 2012 USA	Health Literacy for Older Adults: Using Evidence to Build a Model Educational Program; single arm	Older people (n varied between 11 and 17) Design of workshop topics identified through needs assessment.
Blanson Henkeman 2008 USA	Usability of an adaptive computer assistant that improves self-care and health literacy of older adults; single arm	Older adults (*n* = 28) Evaluation of usability
Coughlan 2012 Ireland	The importance of health literacy in the development of ‘Self Care’ cards for community pharmacies in Ireland; single arm	Patients (*n* = 199) Evaluated self-care cards for 10 ailments
Dwamena 2009 USA	Teaching medical interviewing to patients: the other side of the encounter; single arm	Patients (n varied between 1 and 22) Design-to convert a medical student curriculum for medical interviewing for patients’ use
Eckman 2012 USA	Impact of health literacy on outcomes and effectiveness of an educational intervention in patients with chronic diseases; RCT	Patients (n = unclear) Evaluate pilot test outcomes questionnaire
Ferreira 2005 USA	Health care provider-directed intervention to increase colorectal cancer screening among veterans: results of a randomized controlled trial; RCT	Older people (n = unclear) Design of the patient intervention to be administered by healthcare providers in two outpatient firms.
FØrland 2013 Sweden	Implementation of a Standardised Health Education in a local context. A case study; single arm	Peers (n = 3) Management-sharing their experiences in health education programmes
Gray 2010 USA	Low health literacy as a barrier to medication adherence in patients with diabetes; single arm	Patients (*n* = 49) Evaluation of the project and written educational tool in easy-to-read language
Goeman 2013 Australia	Educational intervention for older people with asthma: A randomised controlled trial; RCT	Older people (n = unclear) Design of Patient Asthma Concerns Tool (PACT)
Kagawa-Singer 2009 USA	Outcomes of a breast health project for Hmong women and men in California; not-RCT	Hmong men and women, key community workers, advisory boards (n = unclear) Design of the culturally specific intervention-flipchart, video, and brochure Hmong women (*n* = 354) intervention
Long 2012 UK	Enhancing health literacy and behavioural change within a tele-care education and support intervention for people with type 2 diabetes; single arm	Patients (*n* = 156) Evaluation only pre/post questionnaire and interviews about blood sugar control on selected sample.
Mayberry 2011 USA	Bridging the digital divide in diabetes: Family support and implications for health literacy; single arm	Patients (*n* = 75) Evaluation of website
Murray 2007 USA	Pharmacist intervention to improve medication adherence in heart failure: A randomized trial; RCT	Patients (n = unclear) Design patient centre instructions for the intervention Patients (314) intervention
Pomerantz 2010 USA	Connecting for health literacy: health information partners (HIPs); single arm	Health Information Partners (HIPS) (*n* = 24) and Minority groups (*n* = 91) Design, management and evaluation of the outreach activities use
Poureslami 2012 Canada	Effectiveness of educational interventions on asthma self-management in Punjabi and Chinese asthma patients: A randomised controlled trial; RCT	Patients, family and community groups (n = 35) Design of educational material Patients (*n* = 40) Evaluation of educational material
Schulz 2010 Switzerland	Coping with chronic lower back pain: Designing and testing the online tool ONESELF; single arm	Patients (n = 15) Design of website Patients (*n* = 748) Management and evaluation
Valle 2006 USA	Fotonovelas: A health literacy tool for educating Latino older adults about dementia; single arm	Members of the Alzheimer’s Association (n = unclear) Design of tool Ethnic older adults (*n* = 111) Evaluation
Welch 2010 USA	Merging health literacy with computer technology: self-managing diet and fluid intake among adult haemodialysis patients; single arm	Renal patients (n = 40) Design and evaluation to confirm previous findings and create their own interfaces
Williams 2013 USA	Kin KeeperSM: Design and baseline characteristics of a community-based randomized controlled trial promoting cancer screening in Black, Latina, and Arab women; RCT	Ethnic women (*n* = 514) Design-recruitment Community stakeholders (n = unclear) Design of studies, test and validate measure implementation of intervention

Note: RCT Randomised controlled trial, single arm no comparator group described, not-RCT Multi-arm non-randomised trial (active and control arms, but not RCT format)

### Demographic details

Thirteen studies were conducted in the USA [29–41] and one in Canada [42]. Three studies were conducted in Europe [43–45], two in the UK [46, 47] and one in Australia [48]. Most studies (*n* = 12) focused on patients with a range of chronic conditions and four on ethnic groups who had immigrated to America [35, 40–42]. Three studies also included demographic variables about low income or low socio-economic status [32, 37, 47]. All the studies included older people, however, six studies were aimed specifically at people over 50 years of age [29, 30, 33, 41, 46, 48].

### Patient and public involvement

The number of PPI representatives in the 20 studies (Table 2) ranged from 3 [44] to over 5000 [46]. Eight studies variously included PPI participants at different stages of research [35, 37, 38, 40–42, 45, 46]. Two studies did not report the number of PPI representatives [32, 33].

**Table 2:** Number of PPI representatives in the studies in the mapping review

No. of participants	1–20	21–40	41–60	61–80	81–100	100 +	Unclear
Number of studies	4	5	1	1	1	9	8

Note: Many studies reported differing numbers of representatives at different stages of the research, thus were added to several categories. Please see Table 1 for full details

Much of patient and public involvement in the 20 studies on health literacy was concentrated into two main domains, design and/or evaluation of the research (Table 3). None of the studies involved patients or the public to identify or prioritise areas or concerns for research, although Aspinall et al. [29] conducted a needs assessment to identify topics for workshops to improve the health literacy of older people. In design, some studies used PPI to develop educational material in various forms and for different uses [29, 31, 33, 37, 43]. Three studies used PPI to inform development of websites [30, 39, 45] and another to educate marginalised groups on the use of health websites [38]. PPI was used to address the unmet health education needs of ethnic minorities [35, 40–42], by enlisting the help of community groups to design interventions that addressed cultural and language barriers. One study enlisted PPI in recruitment [40] and one developed a questionnaire to assess unmet needs of patients [48].

**Table 3:** Number of studies employing PPI in studies on health literacy in different parts of the research process

Domain:	Identify/ prioritise	Design	Grant develop	Undertake/Manage	Analysing/ interpret	Dissemination	Monitoring/ evaluation
Number of studies	0	15 (75%)	0	4 (20%)	0	0	12 (60%)

Note: Several studies reported participants at different stages of the research, thus were added to more than one category. Please see Table 1 for full details

In the undertaking and/or management of research, PPI was active in assisting with educational classes [37, 46], counselling [47], sharing experiences of illness [44], improving cancer screening [33] and disseminating information into communities [38]. Other studies found that information learned through community events was also disseminated to other community and family members [35, 41].

Six studies in the USA [29, 31, 33, 35, 37, 40], and one in Australia [48] used PPI in design only.

A European study included PPI for management only [44]. PPI was used only for evaluation for four studies in the USA [30, 32, 34, 36], one in Europe [43] and one in the UK [47] . Two American studies [39, 41] and one Canadian study [42] used PPI for design and evaluation purposes. Three studies, one in USA [38], one in Europe [45] and on in UK [46] used PPI in all three areas (Table 4).

**Table 4:** Number of studies by country and domains where PPI was used in the research process

Domain		1.Design	2.Management	3.Evaluation	1 & 3	1, 2, & 3
Studies per country	USA	6		4	2	1
	Canada				1	
	Europe		1	1		1
	UK			1		1
	Australia	1				

All studies included research participants aged over 50 years, but only six studies explicitly targeted older people [29, 30, 33, 41, 46, 48]. Advisory and community groups were involved in six studies [35, 38, 40–42, 46]. Minority groups were involved in five studies [35, 38, 40–42], all of which were conducted in USA (Table 5).

**Table 5:** Number of studies with stated types of PPI representatives per country

Who was involved		Volunteers/ peers	Older people	Patients	Community/ advisory groups	Minority groups	Family members
Studies per country	USA		4	5	4	4	1
	Canada			1	1	1	1
	Europe	1		3			
	UK	1	1	1	1		1
	Australia		1				

Note: Several studies reported differing numbers of PPI representatives at different stages of the research. Please see Table 1 for full details

Patients with relevant chronic health conditions were common representatives in research that included PPI. Most studies were conducted in the USA, where patients were involved with design [31, 37] or evaluation of intervention tools [32, 34, 36]. In Europe patients evaluated self-care cards [43], assisted in research management by sharing their experiences in health education programmes [44] and were involved in design, management and evaluation of a website for self-help chronic lower back pain [45]. Patients, along with all the other groups assisted with design and evaluation of self-help educational material for Punjabi and Chinese asthma sufferers living in Canada [42]. American older people contributed to design [29, 33] and evaluation [30] of interventions. Another study included older people and Alzheimer’s Association members in design and evaluation of an educational tool about dementia for Spanish-speaking older Latinos [41]. Older people in Australia assisted with design of a tool to assess concern about asthma [48]. In the UK, older people, together with participants from most other groups (community/volunteers/family) contributed to design, management and evaluation of a fitness intervention to improve health behaviour [46]. Three more American studies included minority (ethnic and lower socioeconomic) group members, two in design of cancer interventions [35, 40], and one for design, management and evaluation of health promotion that also included an advisory group [38]. In one European study peers spoke of experiences of their health conditions to people with similar conditions [44]. One study engaged family members in the development of ethnically and culturally suited educational material for asthma self-management [42]. However, family members were cited by older people as important sources of support for lifestyle changes [46] and for understanding medical information [29]. Interestingly it was not until the evaluation stage that one study found out how important families had been in helping patients use websites to improve health literacy [36] (Table 5).

## Discussion

The aim of this mapping review was to consider when and how PPI activity has been reported in the research process within health literacy studies. A mapping review is an appropriate method where evidence is known to be difficult to find and describe, also when the conceptual definitions and boundaries are relatively new or unclear [25, 49]. A mapping review enabled us to consider some emerging evidence in the nascent and wide ranging field of health literacy intervention research.

Twenty diverse studies were found that reported PPI in the research process. Most studies were conducted in the USA where many addressed health literacy of minority groups. There appears to be a large gap in knowledge about health literacy for minority groups in Europe and other developed countries. PPI was used minimally in most studies. Interventions in Europe (including UK) were more likely to use PPI more comprehensively than those in the USA. Most of the studies in the USA involved PPI at only one stage of research, usually design or evaluation. For example, Aspinall conducted focus groups to identify needs assessment to build an educational program to improve health literacy [29] and in another study patients were recruited to evaluate a website designed to help with diabetes care [36]. Mayberry’s intervention did little for health literacy and the researchers realised the importance of support given by family members only on evaluation. This example highlights the need for PPI to be inclusive of stakeholders that are involved early in the research process. In contrast, Schulz included patients in design, management and evaluation of a website offering self-help to those with chronic back pain [45]. Interestingly, Long only involved patients with diabetes for evaluation of the intervention. However, the intervention was successful in improving knowledge, behavioural and health outcomes, because telecare call handlers provided time and space for patients to develop rapport and ask questions about their condition and related subjects [47]. Patients found it more helpful hearing about living with a chronic condition than about the diagnosis from peers [44] demonstrating the importance of understanding what people want and need to know.

Interpersonal relationships and communication issues throughout the health system are important factors to enable patients to understand their health conditions [17]. Many patients are confused by the language used in medicine and further hindered by ill-health that drains them of energy, although others may become experts on their condition over time [17]. Health literacy affects patients’ and carers’ ability to actively take part in their healthcare [3]. However, few people have any concept of health literacy and for many the term reflects academic language acquired in higher education [17]. Understanding the needs of people to improve their health should drive health literacy interventions. Reflections reported in some of the studies showed that interventions addressed unmet needs [47, 48], improved patients’ self-confidence and ability to communicate with medical professionals [45], and that understanding cultural values had educated a community about dementia [41]. These examples show the wide-ranging nature of the concept of health literacy and suggest that high quality PPI is required to understand and address people’s needs, which in turn, could play an important role in improving health literacy and other interventions [17, 24, 50].

The studies included in our review showed no evidence of PPI living up to the ‘gold standard’ (e.g., GRIPP2) covering the core six principles proposed by Wilson et al. [20] and Staniszewksa et al. [51]. For example, no study reported formally on having independent patient or public members to sit alongside the researchers and feed into the research process. Most studies that included PPI did so at design stage, but none of the studies apparently elicited input at the inception of the research idea. A key finding was that overall PPI was often poorly reported, which aligns with results found by others [50]. Transparency is a much needed requirement and recommended as a national standard for PPI [52]. Lack of transparency and poor reporting reduces propensity to replicate the research, evaluate the actual usefulness of PPI and to explain the factors influencing the outcomes. In some studies patient representation was weak as it was only used for evaluation [53]. Practical problems linked to PPI input to research development were sometimes discussed. For instance Coughlan et al. [43] used members of the Pharmaceutical Care Research Group (University of Cork) to develop self-care cards. These were found on evaluation to be written at a literacy level that was too high for patients to understand [43]. In a UK lifestyle change programme [46], volunteers were comprehensively involved in various aspects of the intervention including delivery, and gained confidence and skills. However, practical issues arose affecting the fidelity of the intervention including misaligned priorities between stakeholders. Similarly, one report described that too much information, often confusing, was put on a website developed with existing patients [45]. These practical issues highlight the need for PPI at the earliest level of research planning, which should be carefully monitored throughout, to ensure that PPI continues to be effective. These gaps and weaknesses should be remediated in future by the GRIPP2 guideline for development of tools to improve reporting of PPI, to understand the context, process, and impact of PPI for better conceptualization or theorization [51].

### Strengths and limitations

This review represents a small subsample of a larger systematic survey of studies on health literacy interventions for older people. Potentially, using an existing inventory could result in studies being missed due to the inclusion criteria of that study not fully reflecting the purpose of the present review. For example, studies for the large inventory used for this review had to report outcomes, thus studies that may identify associations or other important issues around PPI were rejected [26]. Nevertheless, the studies were widely sourced and representative of the existing health literature for older people and the research raised concerns that could be addressed in a dedicated review on health literacy. We relied on screening abstracts at some stages which may have limited our samples; we do not pretend to have undertaken a thorough assessment exercise. We did not assess the intrinsic methodological quality of the studies included in the mapping exercise, neither were we able to compare studies reporting PPI with those that did not.

We note that sometimes PPI representatives seemed to have blurred roles, such as also acting as research subjects [32, 39], or co-investigators [38]. Blurring or dual roles is undesirable because it creates conflicts of interest; the PPI representatives may have cause and opportunity to bias outcomes. A more rigorous review than ours might exclude reports where roles appear to have been blurred.

Our choice of defining older adults as people aged 50+ is inevitably somewhat arbitrary. There is no universally agreed threshold for identifying relatively older adults. Age 50+ does reliably denote older adults as it is more than half of the average lifespan. For public health interventions, an “older adult” threshold at about 50 years may be desirable because this is a potential key window of opportunity to promote health literacy skills that might persist into retirement age, just before health literacy most declines and health care needs are likely to increase.

Production of this article itself did not directly involve patients or public representatives – although consultations with such groups did inform the wider project that led to the creation of the larger inventory that was the starting point for our mapping review [18, 26] . Within our selected studies, PPI was poorly reported. We checked for but failed to find PPI in earlier published developmental work, although we did not contact authors for more information. Many of the studies discussed here pre-date increasingly widespread obligations from funding bodies to orientate research design using PPI. Thus, this mapping review may be considered prescient or premature. Nevertheless, it is illuminating to map the gaps and deficiencies in existing research designs so that appropriate steps can be taken in future research and policy decisions.

## Conclusions

Better reporting is required if the full potential value – and practical issues – of patient and public involvement in the research process are to be understood. Few of the health literacy studies in our review demonstrated patient and public involvement as integral to the research process adopted. Adding PPI to research can enhance quality and appropriateness of the research at every stage, but also holds cultural and ontological challenges for researchers used to being in control. For best effect, PPI should be included at the outset and continue throughout the research. High quality PPI can help develop the evidence base and inform practice for future interventions to improve health literacy among older people.

## Abbreviation

PPI: Patient and public involvement
